# Chorein deficiency promotes ferroptosis

**DOI:** 10.1002/2211-5463.13870

**Published:** 2024-11-08

**Authors:** Yoshiaki Nishizawa, Hitoshi Sakimoto, Omi Nagata, Natsuki Sasaki, Yuka Urata, Kaoru Arai, Hanae Hiwatashi, Izumi Yokoyama, Shosei Kishida, Akira Sano, Masayuki Nakamura

**Affiliations:** ^1^ Department of Psychiatry Kagoshima University Graduate School of Medical and Dental Sciences Japan; ^2^ Department of Biochemistry and Genetics Kagoshima University Graduate School of Medical and Dental Sciences Japan; ^3^ Kagoshima University Japan

**Keywords:** chorea‐acanthocytosis, chorein, ferroptosis, ferrous iron, glutathione peroxidase 4, *VPS13A*

## Abstract

Ferroptosis is a type of programmed cell death owed to an intracellular accumulation of iron resulting in the generation reactive oxygen species, which in turn can cause peroxidation of plasma membrane lipids and ultimately result in cell death. We investigated the potential involvement of *VPS13A* deficiency in ferroptosis. The *VPS13A* gene encodes for chorein, and its deficiency is a molecular cause of chorea‐acanthocytosis (ChAc), a Huntington‐like disease with neurodegeneration in the striatum. In our previous study, we found male infertility characterized by increased malondialdehyde staining of the spermatozoa in the testes of the ChAc model mice. Thus, in this study we performed metabolome analysis of sperm extracted from the epididymis of the ChAc model mice, which revealed decreased cystine levels, suggesting an association between chorein deficiency and ferroptosis. We then investigated the role of chorein in ferroptosis using *VPS13A* knockdown (*VPS13A*‐KD) HEK293 cells. We found that *VPS13A*‐KD cells displayed a significantly diminished resistance to tert‐Butyl hydroperoxide (tBHP)‐induced lipid peroxidation and cell death compared to control cells, which could be rescued by treatment with ferrostatin‐1. Moreover, *VPS13A*‐KD cells showed Fe(II) accumulation, suggesting an impaired capacity for divalent iron removal. In the cytosolic fraction of *VPS13A*‐KD cells, the protein level of glutathione peroxidase 4 (GPX4) was significantly reduced, suggesting that dysfunction of chorein impairs GPX4 transport, thereby facilitating ferroptosis. These results suggest that ferroptosis may contribute to neurodegeneration in ChAc caused by loss of chorein function.

## Graphical abstract

Ferroptosis may contribute to cell death in chorea‐acanthocytosis caused by chorein deficiency. Chorein is suggested to interact with XK protein and be involved in lipid movement within cell membranes. Chorein deficiency can lead to iron accumulation and reactive oxygen species (ROS), causing lipid peroxidation and cell death. Inhibiting ferroptosis may offer a potential therapeutic approach.

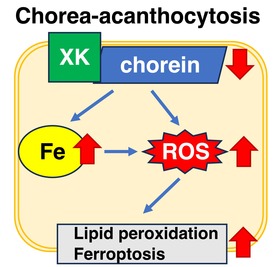

**Abbreviations**
ChAc: chorea‐acanthocytosis
GPX4: glutathione peroxidase 4
HEK: human embryonic kidney
KD: knockdown
NA: neuroacanthocytosis
NBIA: neurodegeneration with brain iron accumulation
PKAN: pantothenate kinase‐associated neurodegeneration
ROS: reactive oxygen species
siRNA: small interfering RNA
tBHP: tert‐butyl hydroperoxide
VPS13A: vacuolar protein sorting 13 homolog A

Ferroptosis is a type of programmed cell death that has recently gained increased attention. In ferroptosis, divalent iron accumulated in the cell generates reactive oxygen species (ROS) [1] that peroxidize plasma membrane lipids, ultimately leading to the disruption of plasma membrane homeostasis and causing plasma membrane destruction [2]. However, divalent iron is essential for cell survival [3], and cells have mechanisms to deal with naturally occurring ROS. Among these, the ROS removal system mediated by cystine/glutamate transporter (xCT) and glutathione peroxidase 4 (GPX4) has attracted much attention. The xCT is a type of antiporter that removes intracellular glutamate and takes in extracellular cystine [4]. The cystine is metabolized to cysteine and then to glutathione, which is used to remove ROS [2, 5]. When the xCT system fails, glutamate accumulates in the cell, while the intracellular level of cysteine and cystine is decreased. GPX4 is a molecule involved in the glutathione‐based ROS removal system [2]. When the GPX4 system fails, the balance between glutathione and oxidized glutathione (GSSG) is disrupted, and the ratio of reduced glutathione/oxidized glutathione (GSH/GSSG) is decreased [6].

In recent years, ferroptosis has been the focus of research as a cause of neurodegenerative diseases [7, 8]. Its involvement in various neurodegenerative diseases such as Huntington's disease [9], Alzheimer's disease [10], and Parkinson's disease [11] has been revealed, and it is a therapeutic target in the treatment of cancer [12].

In humans, loss‐of‐function mutations in the Vacuolar Protein Sorting 13 Homolog A (*VPS13A*) gene, which encodes a large protein named chorein, cause chorea‐acanthocytosis (ChAc). ChAc is a rare neuroacanthocytosis characterized by adult‐onset chorea, acanthocytosis in erythrocytes, and Huntington's disease‐like neuropsychiatric symptoms [13, 14]. The main neuropathological feature of ChAc is neurodegeneration in the striatum [15], but the exact mechanisms leading to neurodegeneration are still unknown. We produced a ChAc model mouse with a homozygous deletion of exons 60–61 (Del/Del mouse) that corresponds to a human disease mutation [16, 17]. The Del/Del mice replicate several key features of human ChAc, including neurodegeneration, behavioral and motor abnormalities, and acanthocytosis. These phenotypic similarities support the use of this mouse model to study the disease mechanisms. Although similar infertility has not been confirmed in humans with ChAc, our previous study demonstrated male infertility characterized by asthenozoospermia and mitochondrial ultrastructure abnormalities in the sperm of Del/Del mice [18]. Recently, Arai et al. [19] showed increased malondialdehyde (MDA) staining in the midpiece region of the spermatozoa of Del/Del mice during spermatogenesis, suggesting the occurrence of ferroptosis in the final stages. To confirm the metabolic changes in sperm from Del/Del mice, we performed a metabolomic analysis of sperm from both wild‐type and Del/Del mice, building upon previous studies [17, 18, 19]. Our preliminary findings provide evidence suggesting the involvement of loss‐of‐function of chorein in ferroptosis. To further explore the relationship between chorein and ferroptosis, we conducted a subsequent analysis of *VPS13A*‐knockdown (KD) human embryonic kidney 293 (HEK293) cells.

## Materials and methods

### Animals

ChAc model mice (Vps13a^tm1asan^ mice) with a homozygous deletion of exons 60–61 in *Vps13a*, corresponding to a human disease mutation, were produced by gene targeting as previously described [16]. We primarily used an inbred strain 129S6/SvEv (wild‐type, WT) (Taconic Labs, Hudson, NY, USA) and the 129S6/SvEv‐Vps13a^tm1asan^ strain (homozygous *Vps13a* exon 60–61 deletion mutation knock‐in, Del/Del), which we produced previously [17, 18]. This study was conducted in adherence to the ethical guidelines for Animal Experimentation (approval number MD23023) and Gene Recombination Experiments (approval number 19S024) as set by the Graduate School of Medical and Dental Sciences, Kagoshima University, Japan. All procedures were approved by the university's ethics review board, ensuring compliance with national and institutional standards for research involving animals and genetic manipulation. Efforts to minimize animal suffering included the use of anesthesia during euthanasia and the implementation of humane endpoints to prevent undue distress. Mice were group‐housed under a normal light–dark cycle (lights on from 7:00 a.m. to 7:00 p.m.) in a clean facility and were given free access to food and water.

### Sampling of mouse sperm

Nineteen‐week‐old WT (*n* = 5) and Del/Del 129S6 mice (*n* = 5) were used for sperm collection. The right and left epididymis were removed from one individual, dissected, and placed in 500 μL of 1 × PBS heated to 37 °C. The tissues were then vortexed for 2 min and centrifuged at 500 ***g*** for 5 min at 4 °C. The supernatant was removed, 400 μL of methanol was added, and the sample vortexed for 30 s. The following steps were performed on ice: 400 μL of methanol solution from each side was collected and pooled in 1.5 mL microtubes, yielding a total volume of 800 μL. Next, 550 μL of ultrapure 1000‐fold diluted Internal Standard Solution 1 (10 mm, HMT) was added, and the mixture was vortexed for 30 s, resulting in a total volume of 1350 μL. One milliliter of the mixture was aliquoted into a new 1.5 mL microfuge tube and centrifuged at 2300 ***g*** for 5 min at 4 °C. The supernatant (350 μL) was transferred to a 5 k ultrafiltration unit (Ultrafree‐MC Plhcc, Sigma‐Aldrich, Burlington, MA, USA) and centrifuged in a swing rotor at 9100 ***g*** for 2 h at 4 °C, with additional 30 min centrifugation at 30 min increments if necessary. The filter cups were removed, the tube lids were closed, cap locks were attached, and the samples were stored at −80 °C.

### Metabolomic analysis of mouse sperm

Mouse sperm samples were sent to Human Metabolome Technologies, Inc (Yamagata, Japan), for metabolomic analysis using Capillary Electrophoresis Time‐of‐Flight Mass Spectrometry (CE‐TOFMS).

### Cell culture and small interfering RNA (siRNA)

The HEK293 cell line was obtained from the Health Science Research Resources Bank (Osaka, Japan). siRNA transfection was performed using Lipofectamine RNAiMAX (13778030; Invitrogen, Carlsbad, CA, USA) according to the manufacturer's instructions.

### Analysis of lipid peroxidation

Approximately 2 × 10^5^ HEK293 cells were seeded on a 12‐well culture plate in Minimum Essential Medium (MEM) and incubated for 16 h with either *VPS13A*‐targeting siRNA or non‐targeting control siRNA. The MEM was then removed, and 1 mL of Hank's Balanced Salt Solution (HBSS) with 1 μL of L248 Liperfluo (Dojindo Laboratories, Kumamoto, Japan) was added to each well. After 30 min of incubation, the HBSS was removed, and 1 mL of HBSS with 0 or 100 μm of tert‐Butyl hydroperoxide (tBHP) (Sigma‐Aldrich) was added to each well. The cells were cultured for 4 h and viewed using a BZ‐X 710 fluorescence microscope system (Keyence, Osaka, Japan). Data analysis and quantification were performed using appropriate software, including the BZ‐X710 application and imagej (U.S. National Institutes of Health, Bethesda, ML, USA).

### Ferrostatin‐1 treatment

The day before the experiment, cells were seeded onto 8‐well slide chambers (80826; ibidi, Bayern, Germany) at a concentration of 10^5^ cells·mL^−1^. The culture medium was removed, and 300 μL of HBSS containing 1 μm Liperfluo was added. After 30 min of incubation at 37 °C, the medium was removed and replaced with 300 μL of HBSS containing 125 μm tBHP and/or 10 μm ferrostatin‐1. After 4 h of incubation at 37 °C, the cells were observed under a fluorescence microscope.

### Cell viability assay

The culture medium was aspirated, and cells were gently washed with 300 μL PBS. Trypan blue (0.4%, 50 μL) (Sigma‐Aldrich) was added to the well and incubated for 3 min at room temperature. Excess trypan blue was removed, and cells were visualized under a microscope. Viable cells remained unstained, whereas dead cells appeared blue.

### Analysis of Fe(II) accumulation

Approximately 2.5 × 10^4^ HEK293 cells were seeded in 96‐well culture plates and incubated for 16 h with MEM containing either siRNA targeting *VPS13A* or non‐targeting control siRNA. The MEM was then removed, and 100 μL of HBSS with 0 or 100 μm ammonium iron(II) sulfate hexahydrate (Fujifilm Wako Pure Chemical Corporation, Osaka, Japan) was added to each well. The cells were cultured for 4 h. The HBSS was then removed, and 100 μL of HBSS with F374 FerroOrange (Dojindo Laboratories) was added to each well. The cells were cultured for 30 min and viewed and recorded with the BZ‐X 710 fluorescence microscope system (Keyence), and fluorescence was measured using a microplate reader (Infinite M200; Tecan, Männedorf, Switzerland). The microplate reader automatically took fluorescence readings at multiple points per well and averaged the readings, which ensured consistent and accurate measurements.

### Subcellular fractionation

HEK293 cells were harvested from a 100 mm culture dish and washed once with PBS. The cells were then homogenized in 1 mL of homogenization buffer consisting of 10 mm HEPES–KOH (pH 7.4), 0.05 m sucrose, 0.22 m mannitol, 1 mm EDTA, and 1 mm PMSF. A whole‐cell extract was obtained by homogenizing the cells with 20 passes in a 27 G needle.

Following this, the homogenates were centrifuged at 800 ***g*** for 10 min at 4 °C to obtain the 0.8 K pellet, which contained the crude nuclear fraction (nuclei and unbroken cells). The supernatant from the 0.8 K pellet was centrifuged at 2300 ***g*** for 10 min at 4 °C to obtain the 2.3 K pellet containing the crude mitochondrial fraction. The 23 K pellet was likewise obtained from the supernatant of the 2.3 K pellet by centrifugation at 23 000 ***g*** for 15 min at 4 °C, which represents the crude lysosomal and peroxisomal fraction. Finally, the supernatant from the 23 K pellet was centrifuged at 100 000 ***g*** for 1 h at 4 °C using a S55A2 rotor (Hitachi Koki, Tokyo, Japan) to obtain the 100 K pellet containing the microsomal fraction, and the remaining supernatant was designated as the cytosol fraction. These fractions were subsequently analyzed by immunoblotting.

### Antibodies

Rabbit polyclonal anti‐chorein antibody (HPA021662) was obtained from Atlas Antibodies (Stockholm, Sweden), goat polyclonal anti‐glyceraldehyde 3‐phosphate dehydrogenase (GAPDH) antibody (sc‐20356) was obtained from Santa Cruz Biotechnology (Dallas, TX, USA), and rabbit monoclonal anti‐GPX4 antibody (ab125066) was obtained from Abcam (Cambridge, UK). Species‐specific horseradish peroxidase‐linked anti‐rabbit IgG from GE Healthcare (Little Chalfont, UK) was used as a secondary antibody.

### Western blot analysis

Protein concentration was determined using the Bradford protein assay (Bio‐Rad, Hercules, CA, USA). Approximately 2.5 μg of protein was denatured in NuPAGE LDS sample buffer (Invitrogen), separated on NuPAGE 4–12% Tris‐acetate gels (Invitrogen), and then electrophoretically transferred to polyvinylidene difluoride (PVDF) membrane (Amersham Biosciences, Little Chalfont, UK). Equal protein loading was confirmed using the Memcode Reversible Protein Stain Kit (Pierce, Rockford, IL, USA). Membranes were blocked using 3% non‐fat dried milk reconstituted in PBS containing 0.1% Tween 20 (PBS‐T) for 1 h at room temperature, then incubated with primary antibodies (anti‐chorein antibody [1 : 1000], anti‐GAPDH antibody [1 : 200] or anti‐GPX4 antibody [1 : 1000]) in PBS‐T for 1 h at room temperature. After rinsing in PBS‐T, the membranes were incubated with a peroxidase‐conjugated, anti‐rabbit antibody (1 : 10 000) for 1 h at room temperature. Proteins were visualized by the ECL Plus Western Blotting Detection System (Amersham Biosciences), and the images were recorded by a digital analyzer (Fujifilm LAS‐1000).

## Results

### Metabolomic analysis

The results of the metabolomic analysis of sperm are presented in Table 1. Sperm samples from WT and Del/Del mice were analyzed. We found that a variety of amino acids accumulated in the sperm of Del/Del mice, while cysteine was below the assay detection limit. Cystine, a precursor of cysteine, was detected in the WT mice, but was below the assay detection limit in the Del/Del mice. Moreover, the levels of both reduced glutathione (GSH) and oxidized glutathione (GSSG) were increased in Del/Del mice compared to WT mice. However, the GSH/GSSG ratio, which is an indicator of the ROS removal system, decreased from 0.48 to 0.30 in Del/Del mice, suggesting impaired ROS scavenging ability. These results suggest that the loss‐of‐function of VPS13A/chorein is involved in ferroptosis. To further investigate the relationship between VPS13A/chorein and ferroptosis, we conducted a subsequent analysis using *VPS13A*‐KD HEK293 cells.

**Table 1:** Metabolomic Differences between Wild‐type (WT) and ChAc model Mice (Del/Del) Sperm. B.D.L., below the assay detection limit; N.A., not available; *p*‐value, from Welch's *t*‐test; SD, standard deviation.

Compound name	Relative area				Comparative analysis	
	WT		Del/Del		WT vs. Del/Del	
	Mean	SD	Mean	SD	Ratio	*P*‐value
Cystine	2.5E‐04	3.8E‐05	B.D.L.	B.D.L.	1<	N.A.
Glutathione (GSSG)_divalent	2.1E‐03	1.2E‐03	5.3E‐03	3.2E‐03	0.39	0.085
Glutathione (GSH)	9.9E‐04	N.A.	1.6E‐03	1.0E‐03	0.62	N.A.
Val	4.3E‐03	1.3E‐03	9.1E‐03	1.1E‐03	0.48	0.001
Leu	3.3E‐03	1.0E‐03	6.7E‐03	1.1E‐03	0.49	0.001
Lys	2.0E‐03	4.8E‐04	4.4E‐03	9.1E‐04	0.44	0.002
Ile	1.9E‐03	6.9E‐04	3.8E‐03	8.8E‐04	0.50	0.005
Met	5.4E‐04	8.2E‐05	1.0E‐03	2.1E‐04	0.54	0.006
Arg	1.5E‐03	2.5E‐04	3.0E‐03	7.0E‐04	0.49	0.006
Pro	2.4E‐03	7.0E‐04	5.4E‐03	1.5E‐03	0.45	0.007
Ala	4.1E‐03	1.2E‐03	9.0E‐03	2.4E‐03	0.46	0.007
Phe	1.8E‐03	7.3E‐04	3.2E‐03	7.3E‐04	0.55	0.013
Thr	2.4E‐03	7.0E‐04	4.0E‐03	1.0E‐03	0.59	0.019
Asp	2.3E‐03	7.9E‐04	4.2E‐03	1.3E‐03	0.55	0.026
Ser	2.8E‐03	1.5E‐03	5.8E‐03	2.0E‐03	0.49	0.035
Glu	4.8E‐03	1.3E‐03	1.0E‐02	4.0E‐03	0.48	0.039
Trp	3.5E‐04	6.3E‐05	7.1E‐04	2.2E‐04	0.50	0.041
His	7.5E‐04	1.4E‐04	1.5E‐03	5.6E‐04	0.51	0.043
Gly	6.5E‐03	1.0E‐03	1.3E‐02	5.6E‐03	0.49	0.053
Gln	9.1E‐03	2.6E‐03	1.4E‐02	6.8E‐03	0.66	0.198
Asn	5.1E‐04	1.2E‐04	1.1E‐03	5.8E‐04	0.48	0.403

### Reduction of chorein levels in *VPS13A* ‐KD HEK293 cells

**Fig. 1:**
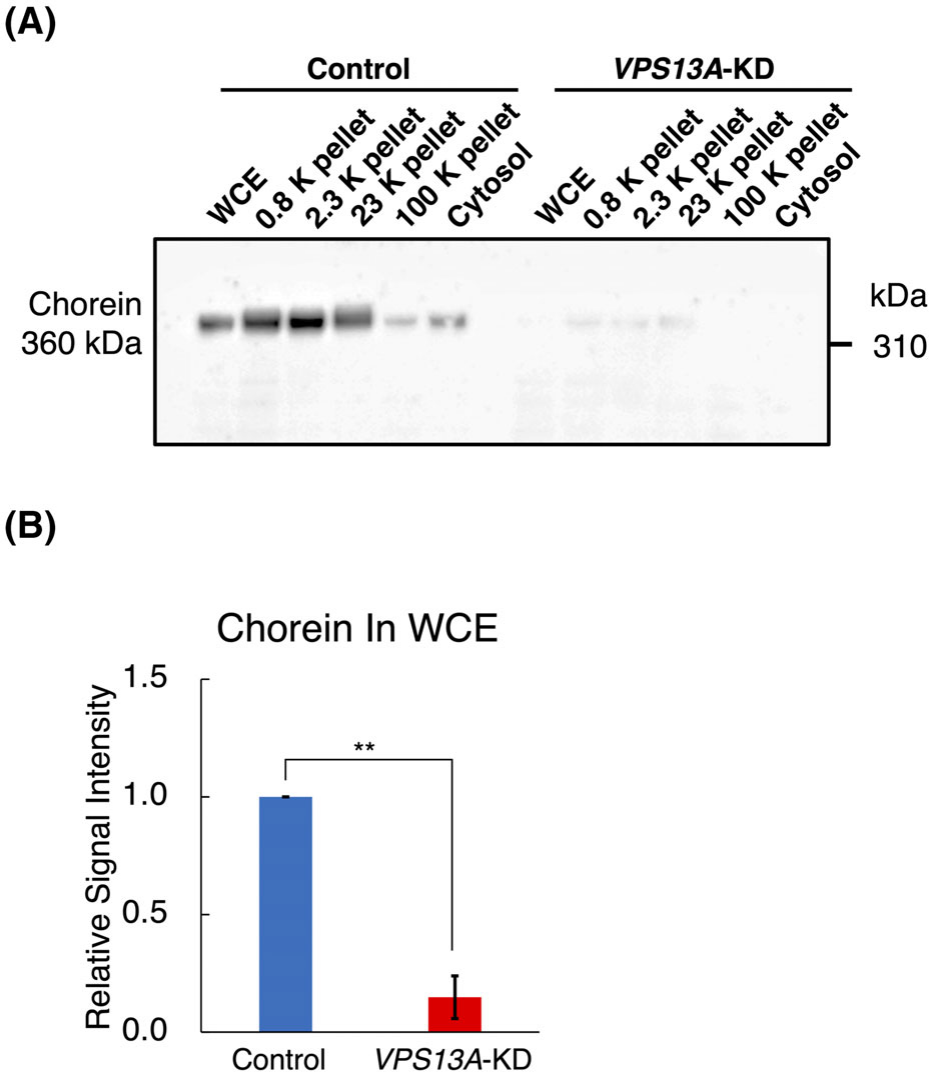
Reduction of chorein levels in *VPS13A*‐KD HEK293 cells. (A) Representative western blot images showing chorein protein levels in whole cellular extract (WCE), 0.8, 2.3, 23, 100 K pellets, and cytosol fractions from si*VPS13A*‐transfected (*VPS13A*‐KD) and mock‐transfected (Control) HEK293 cells. The cellular components of each fraction are as follows: the 0.8 K pellet contains crude nuclear fraction (nuclei and unbroken cells); the 2.3 K pellet contains crude mitochondrial fraction; the 23 K pellet contains crude lysosomal and peroxisomal fraction; the 100 K pellet contains microsomal fraction, and the supernatant from the 100 K pellet is the cytosol fraction (*n* = 5). (B) Quantitative analysis of band density of chorein western blot across the different fractions (*n* = 5). Data indicate a significant decrease in chorein levels in *VPS13A*‐KD cells compared to Control cells. **P < 0.01 (Mann–Whitney *U* tests); error bars represent standard error.

To determine the extent of chorein protein reduction in *VPS13A*‐KD cells, we obtained whole cellular extracts, the 0.8, 2.3, 23, and 100 K pellets, and cytosol fractions from the cell lysates of si*VPS13A*‐transfected HEK293 cells and mock‐transfected control cells. These fractions were then subjected to SDS/PAGE and western blotting with an anti‐chorein antibody. Across all fractions, KD cells exhibited a substantial reduction in chorein levels compared to WT (Fig. 1A,B).

### Plasma membrane lipid peroxidation in *VPS13A* ‐KD HEK293 cells

**Fig. 2:**
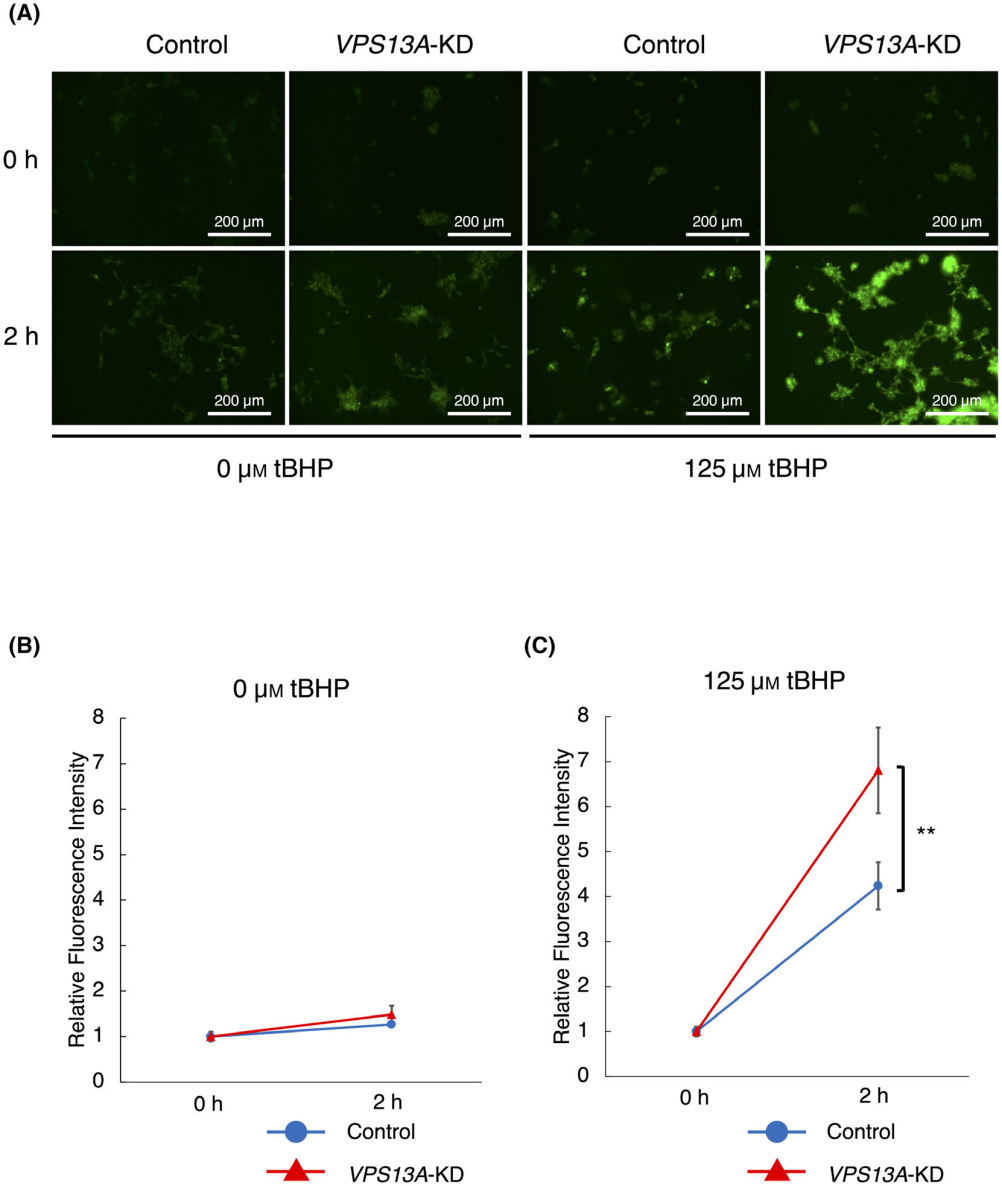
Enhanced lipid peroxidation in *VPS13A*‐KD HEK293 cells upon tBHP stimulation. (A) Fluorescence images of Liperfluo staining in HEK293 cells following stimulation with 0 and 125 μm tBHP, highlighting the difference in plasma membrane lipid peroxides between si*VPS13A*‐transfected (*VPS13A*‐KD) and mock‐transfected (control) cells treated with 125 μm tBHP (*n* = 6). Scale bars represent 200 μm. (B) Quantitative analysis of fluorescence levels in HEK293 cells at 2 h post‐stimulation with 0 μm tBHP shows no differences between control and *VPS13A*‐KD cells (*n* = 6) (Mann–Whitney *U* tests). Error bars represent standard error. (C) Quantitative analysis of fluorescence levels at 2 h following stimulation with 125 μm tBHP shows a significantly higher fluorescence in *VPS13A*‐KD cells compared to control cells (*n* = 6). Data indicate that *VPS13A*‐KD cells exhibit a reduced ability to eliminate lipid peroxide, suggesting decreased resistance to ferroptosis. **P < 0.01 (Mann–Whitney *U* tests); error bars represent standard error.

Subsequently, we aimed to assess the peroxidation of plasma membrane lipids in HEK293 cells. Using Liperfluo staining, a clear accumulation of plasma membrane lipid peroxides following tBHP stimulation was observed in *VPS13A*‐KD cells (Fig. 2). For the experiments, HEK293 cells were stimulated with either 0 or 125 μm of tBHP in HBSS. Fluorescence levels from the WT and KD cells were comparable at 2 h post‐stimulation with 0 μm tBHP (Fig. 2B). However, in the 125 μm tBHP group, fluorescence at 2 h was significantly stronger in the KD cells than in the mock‐transfected control cells (Fig. 2A,C). These results suggest that *VPS13A*‐KD HEK293 cells have a diminished capacity to remove lipid peroxide, indicating a substantial reduction in their resistance to ferroptosis.

### Ferrostatin rescues tBHP‐induced lipid peroxidation and cell death

**Fig. 3:**
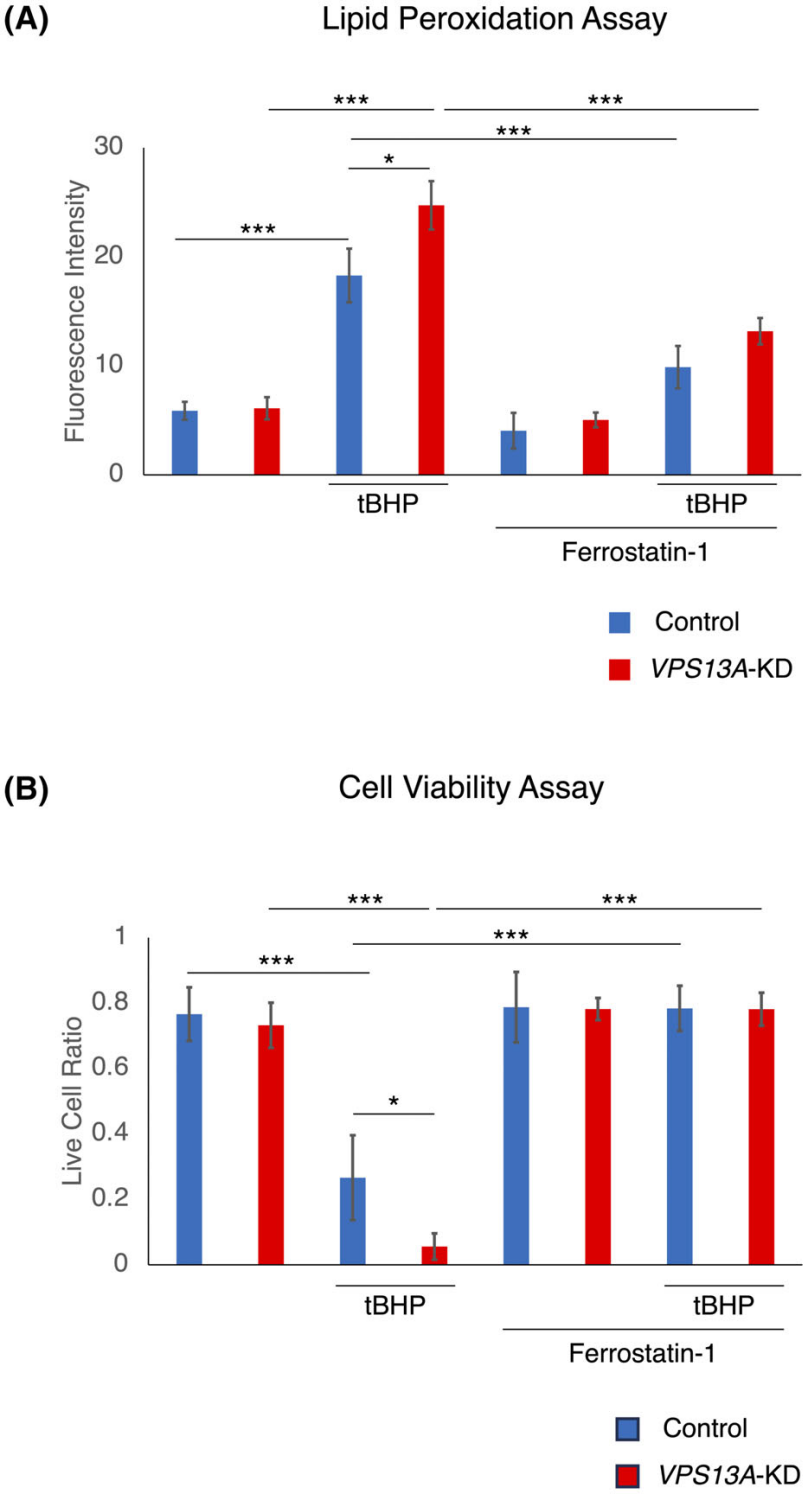
Effects of tBHP and ferrostatin‐1 on lipid peroxidation and cell viability in VPS13A‐KD and control cells. (A) Fluorescence images of Liperfluo staining in HEK293 cells were taken, and fluorescence intensity was measured to assess lipid peroxidation levels. tBHP treatment significantly increased lipid peroxidation in both si*VPS13A*‐transfected (*VPS13A*‐KD) and mock‐transfected (control) cells, with *VPS13A*‐KD cells showing a higher increase compared to control cells. Ferrostatin‐1 pretreatment significantly reduced lipid peroxidation in both tBHP‐treated *VPS13A*‐KD and control cells, eliminating the significant difference between them (*n* = 11). *P < 0.05, ***P < 0.001, (Mann–Whitney *U* tests); error bars represent 95% CI. (B) Cell viability was assessed using the trypan blue exclusion method, measuring the live cell ratio. tBHP treatment significantly decreased cell viability in both *VPS13A‐KD* and Control cells, with *VPS13A‐KD* cells showing lower viability compared to control cells. Ferrostatin‐1 pretreatment significantly increased cell viability in both tBHP‐treated *VPS13A*‐KD and control cells to levels comparable to untreated cells, eliminating the significant difference between them (*n* = 5). *P < 0.05, ***P < 0.001, (Mann–Whitney *U* tests); error bars represent 95% CI.

Both the lipid‐peroxidation assay and the cell viability assay were used to evaluate the effects of tBHP‐induced cytotoxicity and the rescuing effect of ferrostatin‐1 on *VPS13A*‐KD cells (Fig. 3). The lipid‐peroxidation assay showed an increase in lipid peroxidation levels in *VPS13A*‐KD cells treated with tBHP (Figs 2A,C and 3A). This increase in lipid peroxidation was significantly reduced by pretreatment with ferrostatin‐1, indicating a protective effect against tBHP‐induced oxidative damage (Fig. 3A).

Similarly, the cell viability assay, performed using the trypan blue method, demonstrated a significant decrease in cell viability on treatment with tBHP, indicating tBHP‐induced cytotoxicity (Fig. 3B). Pretreatment with ferrostatin‐1 significantly rescued cell viability, suggesting a protective effect against tBHP‐induced oxidative stress (Fig. 3B).

These findings confirm that tBHP induces lipid peroxidation, leading to cell death, and that ferrostatin‐1 effectively rescues cells from both lipid peroxidation and cell death. Both the lipid‐peroxidation assay and the cell viability assay effectively demonstrated tBHP‐induced cytotoxicity and the ability of ferrostatin‐1 to rescue the cells from this cytotoxicity.

### Intracellular Fe(II) changes in *VPS13A* ‐KD

**Fig. 4:**
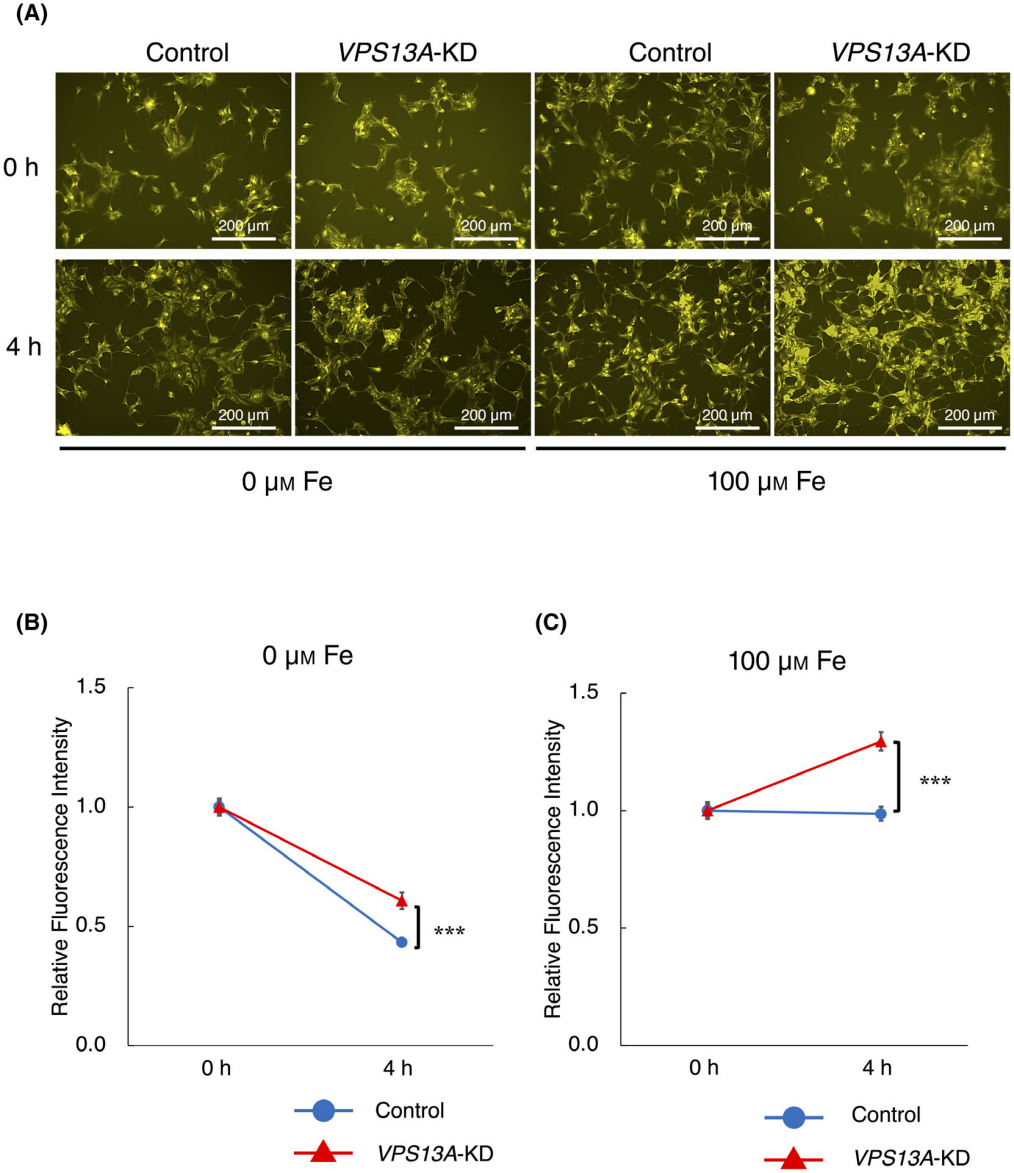
Altered intracellular Fe(II) dynamics in VPS13A‐KD HEK293 cells. (A) Fluorescence images capturing F374 FerroOrange staining of HEK293 cells after treatment with both 0 and 100 μm ammonium iron(II) sulfate, illustrating differences in intracellular Fe(II) between si*VPS13A*‐transfected (*VPS13A*‐KD) and mock‐transfected (control) cells at 0 and 4 h (*n* = 16). Scale bars represent 200 μm. (B) Quantitative analysis of fluorescence levels in cells treated with 0 μm ammonium iron(II) sulfate shows a smaller decrease in fluorescence in *VPS13A*‐KD cells compared to control cells at 4 h (*n* = 16). ***P < 0.001 (Mann–Whitney *U* tests); error bars represent standard error. (C) Quantitative analysis of fluorescence levels in cells treated with 100 μm ammonium iron(II) sulfate at 4 h demonstrates a significant increase in fluorescence in *VPS13A*‐KD cells compared to 0‐h measurements, whereas little change is observed in control cells (*n* = 16). Data suggest impaired Fe(II) efflux in *VPS13A*‐KD cells. ***P < 0.001 (Mann–Whitney *U* tests); error bars represent standard error.

To assess Fe(II) efflux and cellular uptake, intracellular Fe(II) in HEK293 cells was assessed using ammonium iron(II) sulfate in combination with F374 FerroOrange staining. In both the 0 and 100 μm ammonium iron(II) sulfate groups, there was a significant difference in fluorescence between WT and *VPS13A*‐KD cells at 4 h compared to 0 h, with *VPS13A*‐KD cells showing higher levels of fluorescence (Fig. 4). In the 0 μm ammonium iron(II) sulfate group, fluorescence decreased at 4 h; however, less reduction was observed in the *VPS13A*‐KD cells than in the mock‐transfected controls (Fig. 4A,B). In the 100 μm ammonium iron(II) sulfate group, while the control cells showed little change in fluorescence even after 4 h, the fluorescence in *VPS13A*‐KD cells was significantly increased compared to the 0‐h baseline (Fig. 4A,C). These results suggest that the Fe(II) efflux may be impaired in *VPS13A*‐KD cells.

### Reduction of GPX4 in the cytosol fraction of *VPS13A* ‐KD cells (Fig. 5)

**Fig. 5:**
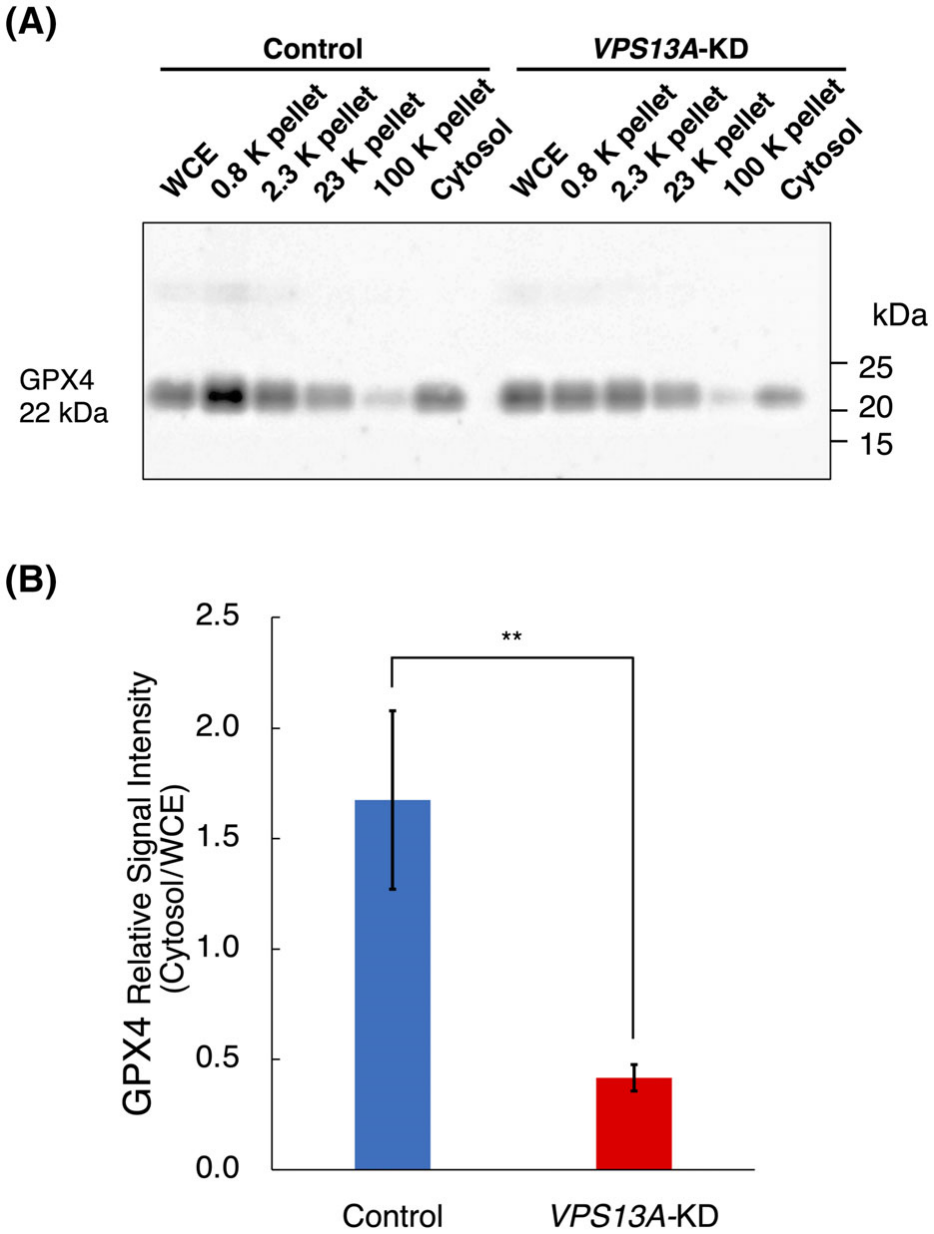
Decreased GPX4 levels in the cytosol fraction of *VPS13A*‐KD HEK293 cells. (A) Representative western blot images of GPX4 in whole cellular extract (WCE), 0.8, 2.3, 23, 100 K pellets, and cytosol fractions from both si*VPS13A*‐transfected (*VPS13A*‐KD) and mock‐transfected (control) HEK293 cells. The cellular components of each fraction are as follows: the 0.8 K pellet contains crude nuclear fraction (nuclei and unbroken cells); the 2.3 K pellet contains crude mitochondrial fraction; the 23 K pellet contains crude lysosomal and peroxisomal fraction; the 100 K pellet contains microsomal fraction, and the supernatant from the 100 K pellet is the cytosol fraction (*n* = 5). (B) Quantitative analysis of GPX4 band density in the cytosol fraction reveals an approximately 75% reduction in *VPS13A*‐KD cells compared to the control (*n* = 5). The overall data suggest a potential role for chorein in influencing the cellular localization of GPX4. **P < 0.01 (Mann–Whitney *U* tests); error bars represent standard error.

To investigate the underlying causes of the decreased ferroptosis resistance in *VPS13A*‐KD cells and the effects of Fe(II) accumulation, whole cellular extracts, the 0.8, 2.3, 23, 100 K pellets, and cytosol fractions were obtained from the cell lysates from both si*VPS13A*‐transfected HEK293 cells and mock‐transfected control cells. These fractions were then subjected to SDS/PAGE and western blotting using an anti‐GPX4 antibody. While there were no significant differences in GPX4 levels between control and KD cells in the whole‐cell extract, the cytosol fraction from KD cells exhibited an approximately 75% reduction in GPX4 levels when compared to the control (Fig. 5A,B). These results suggest that chorein might be involved in maintaining cytoplasmic GPX4 levels.

## Discussion

This study demonstrated that *VPS13A*‐KD HEK293 cells accumulate Fe(II) and are susceptible to iron‐dependent ROS. Similar to conditions in Huntington's disease [20, 21], Alzheimer's disease [22, 23], and Parkinson's disease [24], ChAc neurons may accumulate Fe(II) and be vulnerable to iron‐dependent ROS. This suggests that the neurodegeneration in ChAc could be due to ferroptosis. Additionally, occasional case reports have indicated iron deposition in brain imaging of ChAc patients [25, 26].

The major mechanisms in ferroptosis include the intracellular accumulation of iron and plasma membrane lipids, and possible defense mechanisms include Fe(II) efflux, oxidative conversion of Fe(II) to Fe(III), the removal of plasma membrane lipids, and the removal of ROS.

Chorein itself has a lipid transport function [27, 28], and the XK protein, which binds to chorein, disturbs plasma membrane lipids [29, 30]. Deficiency in XK is known to cause McLeod syndrome, which shares many similarities with ChAc [31]. Both conditions are categorized as neuroacanthocytosis (NA), representing diseases with both neurological disorders and acanthocytosis. A disruption in the molecular system for the removal of plasma lipids may underlie the neurodegenerative features observed in both conditions.

Neurodegeneration with brain iron accumulation (NBIA) presents an intriguing link in this context [32]. This spectrum of disorders, characterized by a progressive movement disorder and iron deposition primarily in the globus pallidus, highlights the relevance of Pantothenate kinase‐associated neurodegeneration (PKAN). PKAN, resulting from the loss‐of‐function in the PANK2 mutation [33], uniquely sits at the crossroads of NBIA and NA syndromes due to the presence of both iron deposition and acanthocytes [32]. As researchers continue to delve into the mechanisms and pathways leading to ferroptosis, its association with diseases like NBIA remains a focal point [34].

The report that chorein is involved in autophagy in mitochondria [35] suggests that mitochondrial dysfunction is involved in iron accumulation. In addition, chorein has been reported to bind to RAB proteins [27, 35]. The possibility of interaction with iron transport proteins also cannot be ruled out. However, since the accumulation of iron accumulation has not been visibly and dramatically altered, it is possible that a non‐iron metal is also involved, or that there is another source of ROS.

In order to understand their subcellular localization and the cellular mechanisms involving chorein and ferroptosis, we performed crude subcellular fractionation followed by western blotting for chorein and GPX4. The present study found that the level of cytosolic GPX4 was significantly decreased in *VPS13A*‐KD cells, which suggests a potential relationship between chorein and the transport of GPX4 to the cytosol. Furthermore, variations observed in GSH/GSSG ratios, combined with the decrease in cystine [2, 4], suggest the possible involvement of GPX4, xCT, and chorein in ROS removal mechanisms. Given that GPX4 is a key molecule in ferroptosis [6], the marked reduction of cytosolic GPX4 in *VPS13A*‐KD cells underscores the relationship between chorein deficiency and susceptibility to ferroptosis.

Ongoing studies are investigating the role of ferroptosis in Huntington's disease [9], Alzheimer's disease [10], Parkinson's disease [11], and other diseases [36]. It is possible that the therapeutic strategies used for these diseases, such as iron chelation therapy, could also be applied to ChAc.

ChAc is characterized by the specific degeneration of cells in the striatum [31], which has been reported to be denervated by ferroptosis in Huntington's disease [9]. Given that ROS play a regulatory role in GABA‐mediated inhibitory neurotransmitter cells [37], which are abundant in the striatum, further studies may provide insight into the mechanisms that underlie the susceptibility of striatal neurons to ferroptosis.

In this study, we elucidated various cellular alterations in *VPS13A*‐KD cells, including excessive lipid peroxidation and cell death due to ROS, iron accumulation, and a decrease in cytosolic GPX4, thereby highlighting a potential relationship between ChAc and ferroptosis at the cellular level. Importantly, we demonstrated that ferrostatin‐1 pretreatment could rescue cells from tBHP‐induced lipid peroxidation and cell death, further supporting the involvement of ferroptosis in ChAc pathology. One limitation of the present study is the use of siRNA‐mediated knockdown of chorein for some experiments. Additional research is essential to extend our understanding of ChAc and its associated pathologies. Future studies should validate our findings in more physiologically relevant models, such as primary cells from Del/Del mice. Investigations using neuronal cells and mouse models can provide crucial insights into cellular mechanisms that are specific to the nervous system and the *in vivo* relevance of these findings. The involvement of chorein in the molecular processes that mediate iron efflux, as well as its potential role in the removal of lipid peroxides, are promising areas of inquiry. It might also be worthwhile to explore how chorein interacts with other cellular pathways and proteins that have not yet been identified, as they could provide additional insight into the etiology of ChAc and potential therapeutic targets. Elucidating the molecular involvement of ChAc in ferroptosis would pave the way for promising developments in drug therapy. Considering the complexities associated with ChAc, a multi‐faceted approach that encompasses diverse research methodologies, including the use of mouse models, will be pivotal in advancing our knowledge and developing effective treatments.

## Conflict of interest

The authors declare no conflict of interest.

### Peer review

The peer review history for this article is available at https://www.webofscience.com/api/gateway/wos/peer‐review/10.1002/2211‐5463.13870.

## Author contributions

YN, AS, and MN contributed to the conception. YN, AS, and MN designed the study. YN, HS, NS, KA, YU, AS, and MN contributed to the interpretation or analysis of data. YN, HH, and IY performed the experimental procedures. YN, HS, ON, HH, and IY contributed reagents, materials, and analysis tools. YU, HS, ON, NS, and MN contributed to the funding acquisition. YN, HH, and IY contributed to the preparation of the original draft manuscript. AS, SK, and MN contributed to the supervision. AS and MN critically revised the manuscript. All authors approved the final manuscript.

## Data Availability

The data that support the findings of this study are available from the corresponding author [nakamu36@m.kufm.kagoshima-u.ac.jp] upon reasonable request.
